# Approaches for Efficiently Detecting Frontier Cells in Robotics Exploration

**DOI:** 10.3389/frobt.2021.616470

**Published:** 2021-02-25

**Authors:** Phillip Quin, Dac Dang Khoa Nguyen, Thanh Long Vu, Alen Alempijevic, Gavin Paul

**Affiliations:** Centre for Autonomous Systems, University of Technology, Sydney, NSW, Australia

**Keywords:** frontier detection, frontier-based exploration, robot exploration, field robotics, mobile robots

## Abstract

Many robot exploration algorithms that are used to explore office, home, or outdoor environments, rely on the concept of frontier cells. Frontier cells define the border between known and unknown space. Frontier-based exploration is the process of repeatedly detecting frontiers and moving towards them, until there are no more frontiers and therefore no more unknown regions. The faster frontier cells can be detected, the more efficient exploration becomes. This paper proposes several algorithms for detecting frontiers. The first is called Naïve Active Area (NaïveAA) frontier detection and achieves frontier detection in constant time by only evaluating the cells in the active area defined by scans taken. The second algorithm is called Expanding-Wavefront Frontier Detection (EWFD) and uses frontiers from the previous timestep as a starting point for searching for frontiers in newly discovered space. The third approach is called Frontier-Tracing Frontier Detection (FTFD) and also uses the frontiers from the previous timestep as well as the endpoints of the scan, to determine the frontiers at the current timestep. Algorithms are compared to state-of-the-art algorithms such as Naïve, WFD, and WFD-INC. NaïveAA is shown to operate in constant time and therefore is suitable as a basic benchmark for frontier detection algorithms. EWFD and FTFD are found to be significantly faster than other algorithms.

## 1 Introduction

The concept of frontiers was first proposed by Yamauchi. (1997). Frontiers have since been used in many robot exploration strategies, whether by single robots (Digor et al. (2010); Quin et al. (2013); Shade and Newman. (2011); Paul et al., (2015); Dornhege and Kleiner. (2011); Paul et al., (2016)) or teams of multiple robots (Faigl and Kulich. (2013); Reid et al., (2013); Hassan et al., (2018)). Since frontier detection is a component of exploration algorithms, speeding up frontier detection will speed up the exploration process. Allowing faster frontier detection may also improve the quality of the decisions made by the exploration algorithms since faster decisions can be made with more recent data. The speed of new sensors means that frontier detection is more likely to be the bottleneck to fast robot exploration and therefore needs to be made more efficient.

Maps are often represented using occupancy grids, or connected regions (Twigg et al., (2013)), in which cells represent a physical location in space. A cell can have several states, it can be “unknown” meaning there may or may not be an obstacle in that cell, it can be “known freespace”, which means there is no obstacle in it and can be safely passed through, or the cell can be “known occupied”. In the rest of this paper “known freespace” and “known occupied” will simply be referred to as freespace and occupied, respectively. This state is usually represented as a value from 0 to 1 representing that cell’s likelihood of containing an obstacle. Frontiers are cells of an occupancy grid that are freespace and which have at least one neighboring cell that has an unknown state (Yamauchi. (1997)). While there exist probabilistic approaches to frontier detection (Liu et al. (2020)), it is out of the scope of this paper when comparing the proposed algorithms with other probabilistic approaches as they use different basic data type structures.

The Naïve approach to detecting frontiers is to evaluate every cell in the robot’s map and determine whether it is freespace and has at least one unknown neighbor. Table 1 shows how slow this approach is in large real-world environments such as the Freiburg corridor environment and Freiburg campus (Wurm et al., (2010)), whether in the 2D or 3D case. In MATLAB, on an Intel i5-6,500 3.20 GHz machine, evaluating whether a cell is a frontier or not takes 12 microseconds. In the four example maps in Table 1, this means evaluating each cell to determine frontiers would take approximately 105.5 s, 1.6 h, 9.6 h, and 27 weeks, respectively.

**TABLE 1:** Example iteration times for Naïve frontier detection if cell evaluations take 1 ms, 1us, or 1 ns? The first two rows are the 2D areas of the “FR-079 corridor” and “Freiburg campus” data sets respectively. The second two rows are the full 3D volumes of the same data sets, Hornung et al., (2013).

Dimensions (m)	${\text{cm}}^{2}$ Cells/${\text{cm}}^{3}$ Voxels	1 ms	1us (s)	1 ns (s)
43.8 × 18.2	$8.79\times 10^{6}$	2.44 h s	8.79	0.01
292 × 167	$4.88\times 10^{8}$	5.6days	8.13	0.49
43.8 × 18.2 × 3.3	$2.90\times 10^{9}$	4.8weeks	48.35	2.90
292 × 167 × 28	$1.37\times 10^{12}$	43.41years	2.25	22.76

Faster algorithms have been proposed such as Wavefront Frontier Detector (WFD) (Keidar and Kaminka. (2012)), Fast Frontier Detection (FFD) (Keidar and Kaminka. (2012)), Incremental Wavefront Frontier Detector (WFD-INC) (Keidar and Kaminka. (2014)), and Incremental-Parallel Frontier Detector (WFD-IP) (Keidar and Kaminka. (2014)). WFD involves beginning with the robot’s current location and performing a Breadth-First Search (BFS) from that position through freespace cells until frontier cells are encountered. This algorithm has the advantage over the Naïve approach of only evaluating the subset of the map that is freespace.

WFD-INC uses the same principle as WFD but bounds the BFS to the active area of the most recent sequence of scans, (i.e. the scans that have occurred since the last call to WFD-INC). In this context, a scan refers to a 2D (or 3D) scan from a sensor such as a laser rangefinder, LiDAR, but the frontier detection principle is equally relevant to a depth camera or stereo vision sensor. The active area of a scan is the bounded area defining the region of the map modified by the scan; the simplest example being a bounding box. By using a bounded area, WFD-INC runs in time proportional to the size of the active area rather than the size of the map. WFD-IP is very similar to WFD-INC but takes advantage of parallel computation.

**FIGURE 1:**
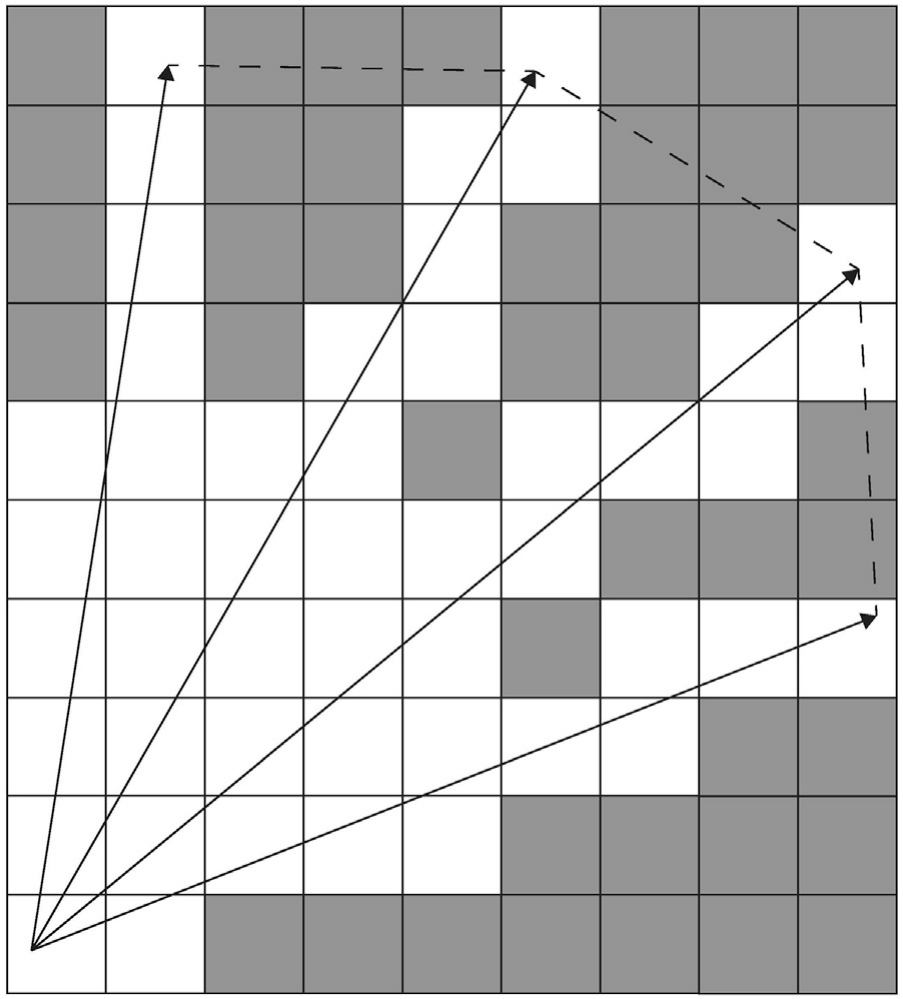
Rays traversing the occupancy grid (white cells are freespace and gray cells are unknown space). Dashed lines show the cells that the Bresenham algorithm would traverse when determining cells on the line between ray endpoints.

Fast Frontier Detection (FFD) by Keidar and Kaminka. (2012, 2014) involves only evaluating the cells in each individual scan, particularly the edges of the scan range, along which any new frontier must necessarily lie. A bounding box is built around the scanned area. Cells that were frontiers in this bounding box before the new scan was obtained are checked to see if they are no longer frontiers at the current time step. This approach is faster than WFD, but requires that frontier detection take place after every scan. FFD is also less likely to correctly detect frontiers at the maximum range of the sensor. Divergence in laser points at extreme ranges means that Bresenham’s line algorithm, which is a method for line tesselation (see Figure 1), and that is used by FFD to determine the contour evaluated for potential frontiers, will cut across unknown space and not cover all cells that should be detected as frontiers (see Figure 1). This makes implementation more complex and restricts the applicability of the strategy to specific sensor ranges and footprints. These calculations may be wasteful for exploration strategies that take many scans before the algorithm recalculates frontiers to decide where to move next (Quin et al., (2016; 2017)).

Senarathne et al. present an algorithm called OBB based Frontier Detector (ODF) (Senarathne et al., (2013); Senarathne and Wang. (2015)). OFD keeps track of the updated cells and their previous states to efficiently update the set of frontier cells. However, when there are a large number of updated cells relative to the edges of the new observation, cells will be evaluated unnecessarily.

Qiao et al., (2018) introduce a frontier detection utilizing the idea behind a randomly exploring random tree. A tree is constructed from the current position of the robot to locate the frontiers in the map, using the customized branching and selection rules. When a set of frontier cells is detected, and the robot passes through that area, all of the tree branches are then removed to free up memory and speed up the calculation process. Orsulic et al., (2019) propose a method of frontier detection for a multi-robot scenario, where each of them performs frontier detection separately in local maps. A global map is then constructed to combine the information from all local maps with all of the frontiers. However, a single robot performing exploration is unable to take advantage of the multi-robot collaborative nature of this detection algorithm.

This paper presents the details of two frontier detection algorithms called Naïve Active Area frontier detection (NaïveAA) and Expanding-Wavefront Frontier Detection (EWFD), which were first introduced by the authors in a conference paper (Quin et al., (2014)). It also provides all new simulated and real-world experimental results and discussions for both algorithms. A novel algorithm for frontier detection, called Frontier-Tracing Frontier Detection (FTFD) is also presented in this paper. The experiments and analysis are performed for a variety of 2D cases. NaïveAA evaluates all cells in the active area of the scan, adding or removing cells from the set of frontier cells as needed. EWFD begins a BFS from previously detected frontier cells in the active area, evaluating only freespace cells that have not yet been evaluated at any previous timestep. FTFD uses the frontiers from the previous timestep and the endpoints of the sensor envelope as starting points for finding the new frontiers at the current timestep. The run-time of all three algorithms is determined theoretically and through simulations, and compared to other state-of-the-art algorithms.

The structure of the rest of the paper is as follows. In Section 2, the nomenclature used in the following description, proofs, and analyses is defined. NaïveAA is then described in Section 3. EWFD is described in Section 4 along with proofs of soundness and completeness and theoretical analysis of best and worst case execution times. FTFD is described in Section 5, which also provides proofs of FTFD’s soundness and completeness and includes theoretical analysis of the best case and worst case execution times of FTFD. Results of simulations and using real-world data are presented in Section 6. Section 7 contains concluding remarks and suggestions for future work.

## 2 Nomenclature

The set of cells in the robot’s map will be *M*. The size of the set *M* is, therefore, |M|. The set of freespace cells in the map, after the environment has been completely explored, is $M_{\text{free}}$. The map is divided into cells which can have several states; unknown, freespace, or obstacle.

The set $P_{t}^{\text{free}}$ is the set of known freespace at time *t*. Similarly, $P_{t}^{\text{obs}}$ is the set of known obstacles at time, *t* and $P_{t}^{\text{unk}}$ is the set of unknown space at *t*.

$O_{t}$ is the sensor observation made at time *t*, and $S\left(O_{t}\right)$ is the set of cells covered, (i.e. visible) by the observation $O_{t}$. $A_{t}$ is the “active area of the observation at time *t*. The active area is an overestimate of the region covered by the sensor field of view made for ease of computation. The simplest definition is an axis-aligned bounding box minimally containing the origin of the sensor observation and the sensor ray endpoints.

The limit to the number of cells observed as part of any single sensor observation is denoted by $S_{\max}$. The corresponding upper limit to the size of the active area is $A_{\max}$, such that $S\left(O_{t}\right)\leq S_{\max}\leq A_{\max}$.

There is a subset of $S\left(O_{t}\right)\underset{x\to \infty }{\lim}$ that comprises of cells that are adjacent to cells that are *not* in $S\left(O_{t}\right)$. This subset forms a boundary or edge, and these cells are denoted $ℰ\left(O_{t}\right)$. The largest size this set could ever be is ${ℰ}_{\max}$.

A cell is said to be neighboring another cell if it is directly adjacent to it, sharing at least an edge or a vertex.

Finally, the set of frontier cells at time *t* is $F_{t}$.

## 3 Naïve Active Area Frontier Detection

The underlying principle of NaïveAA, presented in detail by Quin et al., (2014), but described briefly here for completeness, is that only cells that are in the active area need to be evaluated as having become frontiers or no longer being frontiers. NaïveAA (Algorithm 1), therefore, involves iterating through each cell, *c*, in the active area, $A_{t}$, of any scans taken since the last frontier detection step, and includes the cells immediately adjacent to the scanned areas. Cells that are no longer a frontier are removed from $F_{t}$, whereas cells that are new frontiers are added to $F_{t}$.

As with the other algorithms, Kosaraju’s series of Depth First Searches (KOSARAJU_DFS) is then run to determine which frontier cells are connected in O(|F|) time.

**ALGORITHM 1:**
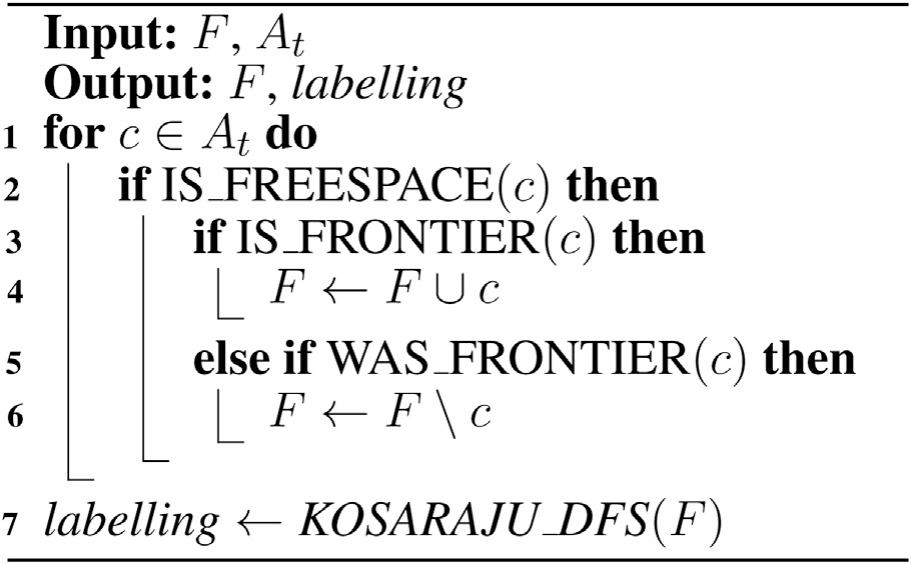
Naïve active area frontier detection (Quin et al., 2014).

## 4 Expanding-Wavefront Frontier Detection

The EWFD algorithm was introduced by Quin et al., (2014), but a compact description is provided here for completeness and for comparison to the novel algorithm, FTFD, being presented in this paper.

The first iteration of the EWFD (Algorithm 2), when there are no known frontiers, is similar to WFD, in that a BFS is performed from the robot’s position. Freespace cells are evaluated to check if they are frontiers and, if they are indeed adjacent to an unknown cell, the cell is added to the frontier set. Freespace cells have their neighbors added to the queue. As each cell is visited by the BFS, it is marked as visited. This labeling is not removed after the EWFD iteration.

Once there exists a set of frontiers, subsequent iterations of EWFD include an added step. Before beginning the BFS, EWFD finds the set of frontiers from the previous timestep that is inside the active area of the latest scan (or sequence of scans). These cells are marked as unvisited and added to the BFS queue. Now, as freespace cells are popped off the queue for evaluation, they are also checked for frontier status. If they are in the frontier set but are no longer frontiers in the current timestep, then they are removed from the frontier set.

Should the robot ever move completely into unknown space, then the space the robot physically occupies without collisions can, as in the initial case, be assumed to be free, and a set of frontiers surrounding it are known as a result. This can be performed through the use of a simple frontier detection approach such as NaïveAA. The sensor, for the purposes of frontier detection, again lies within known space.

After the set of frontiers has been updated, the set of connected frontier groups can be determined by using Kosaraju’s connectivity algorithm, which consists of a series of Depth-First Searches (DFS).

## 5 Frontier-Tracing Frontier Detection

Given an initially unknown environment in which a robot exists, it follows that since the robot occupies physical space, that space is freespace. If there is known freespace, then there is a set of frontiers, $F_{0}$, which can also be known a priori, or easily detected, before exploration begins. Exploration consists of observations, $O_{t}$, made at particular times, *t*, followed by frontier detection which results in a set of frontiers, $F_{t}$.

**ALGORITHM 2:**
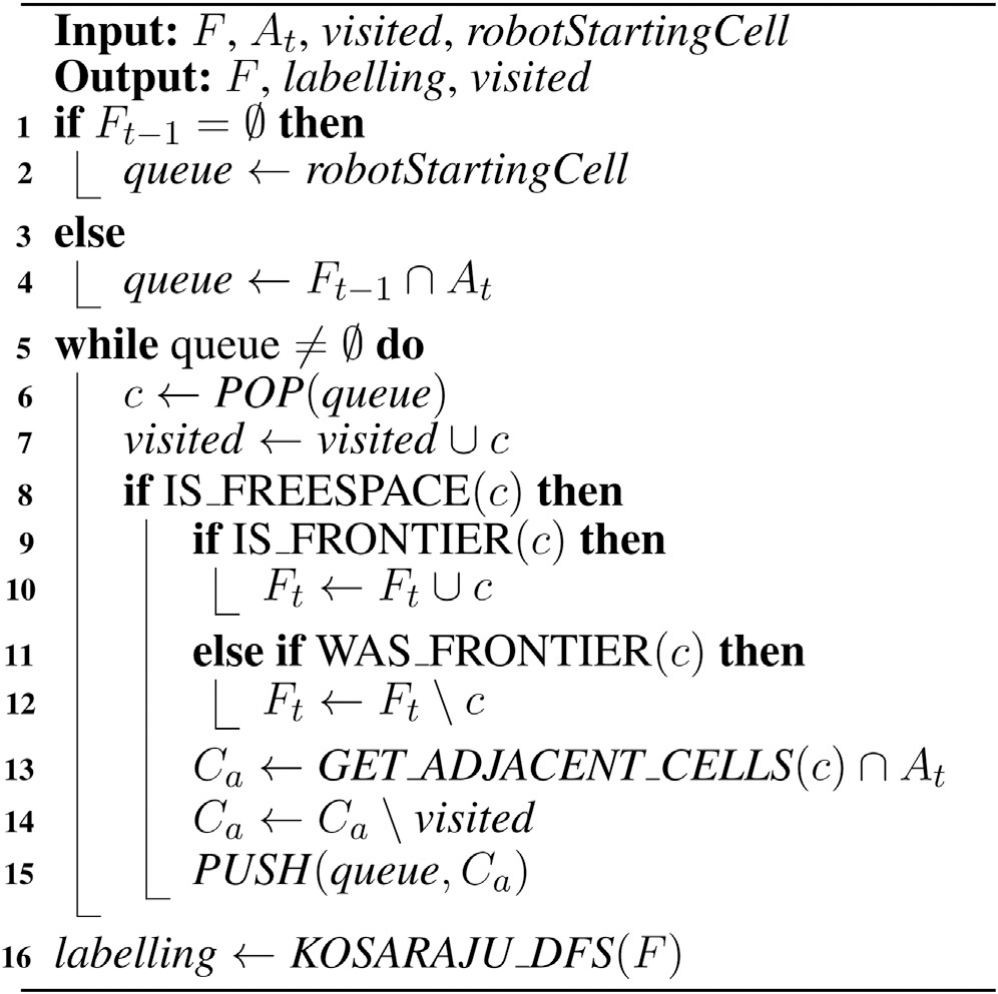
Expanding-wavefront frontier detection (Quin et al., 2014).

During the course of safe exploration, the sensor origin will generally remain inside known freespace. Even in the case of exploration approaches that permit path planning through unknown space (Wettach and Berns. (2010)), sensor observations are taken during robot motion such that the unknown space becomes known space before the robot moves through it.

Should the robot ever move completely into unknown space, then the space the robot physically occupies without collisions can be assumed to be free, and a set of frontiers surrounding it are known as a result, such as by using the simple frontier detection approach, NaïveAA. The sensor, for the purposes of frontier detection, again lies within known space.

If the sensor origin lies on the perimeter or surface of the sensor footprint, (i.e. the field of view (FOV) of the sensor), and if the sensor origin covers some previously unknown space in the sensor’s FOV, then the edges of the sensor footprint intersect with the current known set of frontiers, $F_{t-1}$. If an observation is made at time *t*, the set of obstacles and frontiers newly formed by the observation will intersect with $F_{t-1}$.

Thus, the cells that are now newly part of the frontier as a result of $O_{t}$ will be connected to the old frontier or to obstacles that are connected to the old or new frontier. Obstacles are considered connected to a frontier if they neighbor a frontier cell belonging to that frontier, or neighbor an obstacle cell connected to that frontier.

**FIGURE 2:**
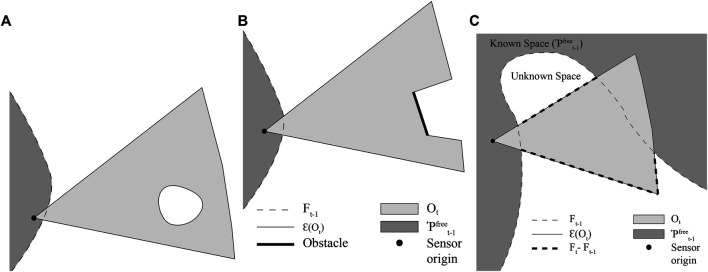
**(A)** An example of a “bubble”, which is not possible; **(B)** an example of how obstacles deform the area covered by $O_{t}$; **(C)** the relationship between frontiers at t−1 and an observation $O_{t}$.

Since we assume the sensor FOV can be modeled as sufficiently dense rays traveling outwards from a single point, it is impossible for a pocket of space to remain unknown and encapsulated in newly discovered freespace (see Figure 2A).

The only case in which the old and new frontiers will not be connected is if at *t*, all known space, and therefore all frontiers, $F_{t-1}$ are in $O_{t}$. This would result in $F_{t}$ being completely disjoint from $F_{t-1}$. For the purposes of FTFD, $F_{0}$ is assumed to be the frontier set existing after sufficient exploration has been performed such that $F_{0}$ cannot be contained inside the sensor footprint.

FTFD (Algorithm 3) uses the fact that the perimeter of the latest observation is highly likely to intersect with the previously known frontiers. The previous frontiers that lie within the observed area, and the sensor ray endpoints, are used as a starting point to search for new frontiers, which lie along the observation’s perimeter. More precisely, an observation is made at *t* and the robot’s map of the environment is updated. The task is now to update the frontier set, $F_{t-1}$ to incorporate the new information contained in $O_{t}$, and to create the set of frontiers, $F_{t}$.

**FIGURE 3:**
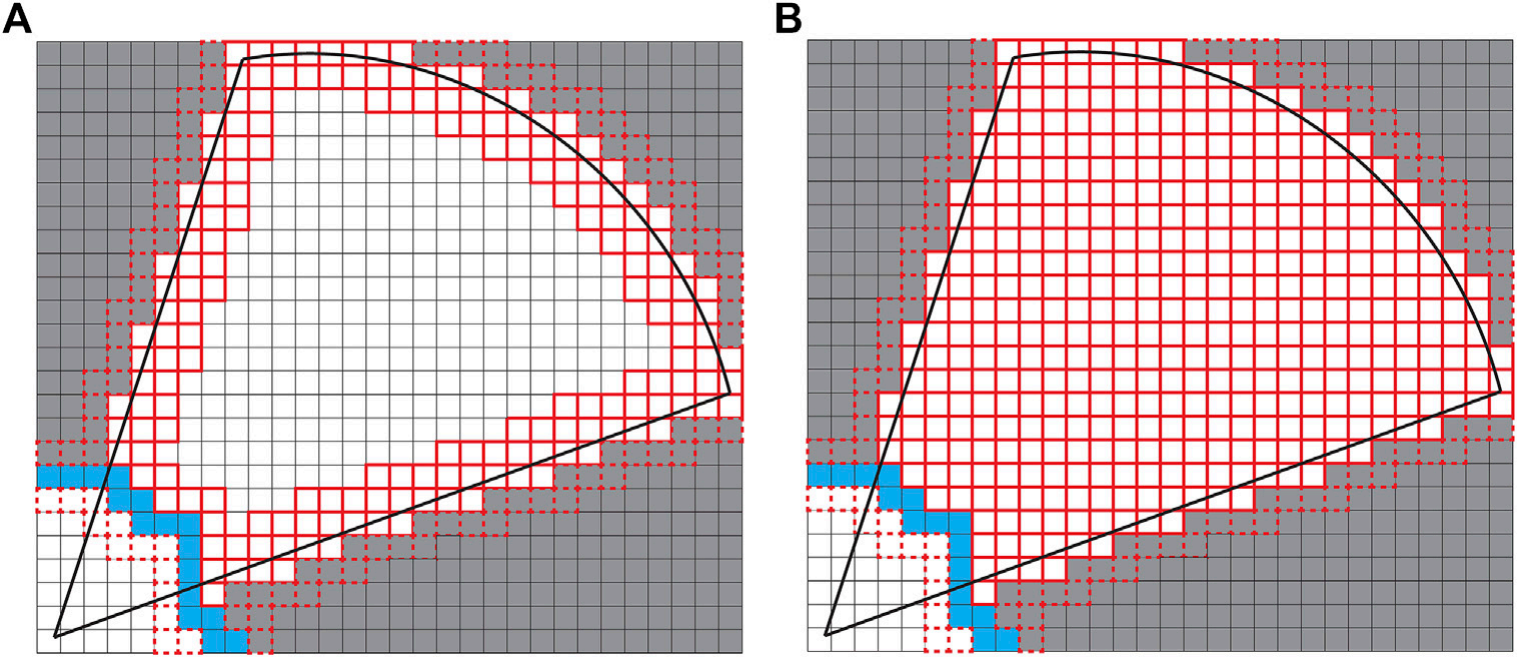
**(A)** FTFD; **(B)** EWFD. Thick red borders are cells that are evaluated as potential frontiers, dashed borders are cells popped from the queue but discarded before full evaluation. Blue cells are frontier cells from the previous timestep, and are also evaluated. Thick black lines denote the sensor FOV.

$F_{t}$ is initially equal to $F_{t-1}$. A set of frontiers, $F_{\text{aa}}$, is created, which is a subset of the frontier cells in $F_{t-1}$ that lie inside $A_{t}$. A BFS is initialized with the active area frontiers, $F_{\text{aa}}$ in its queue, as well as cells intersected by the endpoints of the rays used to integrate the latest scan into the map. At each iteration of the BFS, a cell, *c* is popped off the queue. If *c* is an unvisited frontier, it is added to $F_{t}$, and its neighboring freespace cells that lie in the active area in the map are added to the queue. If *c* is not a frontier, but was a frontier at t−1, then it is removed from $F_{t}$. If *c* is an obstacle, its freespace neighbors are similarly added to the queue. The resulting pattern of cells evaluated is shown in Figure 3A. This pattern for FTFD is compared to that of EWFD in Figure 3B.

Exploration strategies that use frontiers often need connected frontier cells grouped into frontier objects. This is so that the length of a frontier can be determined, or so that a robot can be directed towards the midpoint of a frontier. If such frontier grouping is required, then Kosaraju’s series of Depth First Searches (KOSARAJU_DFS) is performed on $F_{t}$ to determine which frontier cells should be grouped together.

**Algorithm 3:**
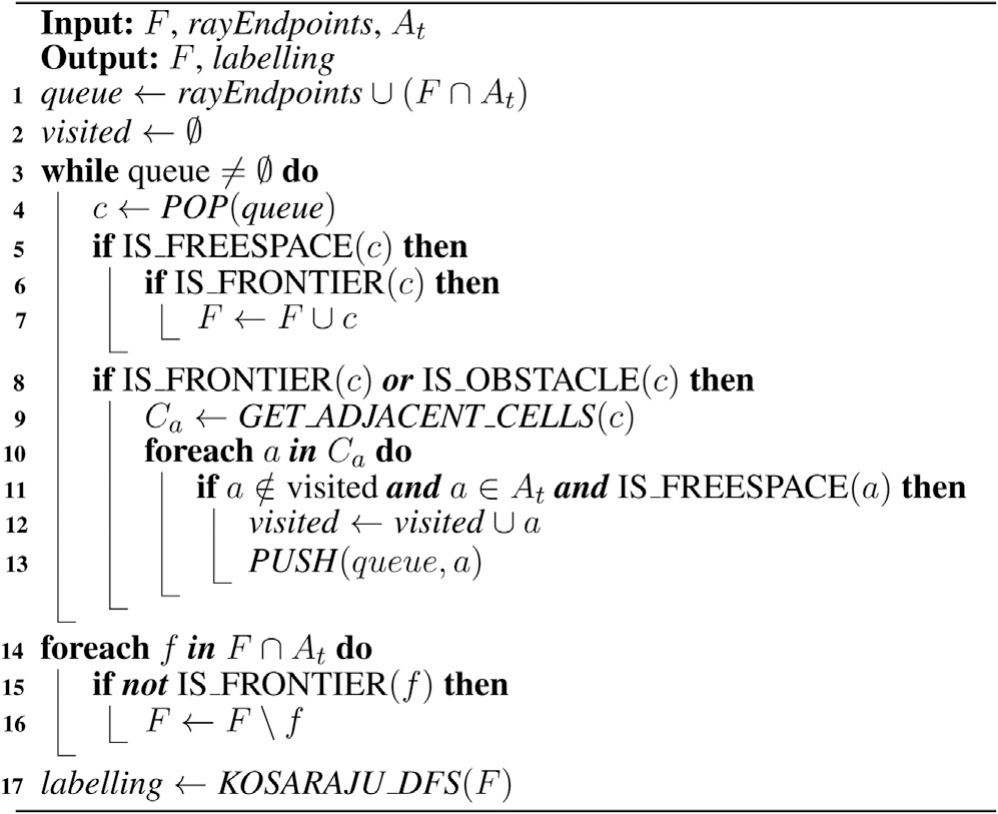
Frontier-tracing frontier detection.

### 5.1 FTFD Soundness and Completeness

It is assumed for FTFD that there are no pockets of unknown space encapsulated within the outer perimeter of the scan after a scan has occurred (see Figure 2A). It can then be proved that FTFD is complete and sound, (i.e. it will correctly identify all frontier cells that delimit the boundary between unknown space and the known space that contains the robot, and it will not mislabel a cell as a frontier when that cell is not the boundary between known and unknown space).

Lemma 5.1. Suppose f is a frontier cell at t, which was not a frontier cell at iteration t−1. Then FTFD will label f as a frontier cell.

PROOF This proof considers the ${\mathbb{R}}^{2}$ case for clarity, the reasoning is equally applicable to ${\mathbb{R}}^{3}$.

Let $O_{t}$ represent the observation made at *t*, and let the cells covered by $O_{t}$ be denoted $S\left(O_{t}\right)$, i.e. the surface or area of $O_{t}$. Let $ℰ\left(O_{t}\right)$ be the “perimeter” of $S\left(O_{t}\right)$, i.e., the cells that are in $S\left(O_{t}\right)$, and are adjacent to cells in the robot map that are not in $S\left(O_{t}\right)$. Let $F_{t-1}$ be the set of frontier cells in the robot map at t−1 that represent the boundary between known physical free space, $P_{t-1}^{\text{free}}$, “inside” the boundary, and unknown space “outside” the boundary. Let $F_{\text{new}}$ denote the set of newly created frontiers that ought to be detected by FTFD, such that $f\in F_{\text{new}}$, where *f* is a frontier cell at *t*, and was not a frontier cell at t−1.

Only cells in $S\left(O_{t}\right)$ will have been changed by $O_{t}$, from their state at t−1. Therefore, only cells in $S\left(O_{t}\right)$ are potentially in $F_{\text{new}}$. Cells in $S\left(O_{t}\right)$ are known, either freespace or obstacles, which means $ℰ\left(O_{t}\right)$ is also composed of either freespace cells, or obstacle cells (see Figure 2B). By definition, a cell in $S\left(O_{t}\right)$ but not in $ℰ\left(O_{t}\right)$ can only be adjacent to other cells in $S\left(O_{t}\right)$, and cannot be a frontier. Therefore, only freespace cells in $ℰ\left(O_{t}\right)$ are potentially members of $F_{\text{new}}$. Furthermore, only freespace cells in $ℰ\left(O_{t}\right)$ that are not in $P_{t-1}^{\text{free}}$ and not in $F_{t-1}$ can be new frontiers, since any freespace cell inside known space that is not already a frontier in $F_{t-1}$ can only be adjacent to known space. Finally, since all freespace cells in $ℰ\left(O_{t}\right)$ must be adjacent to a cell not in $S\left(O_{t}\right)$, then all freespace cells in $ℰ\left(O_{t}\right)$ that are not in $P_{t-1}^{\text{free}}$ and not in $F_{t-1}$ must be new frontier cells, since they will be adjacent to unknown cells.

In order for new frontiers to exist at *t*, $S\left(O_{t}\right)$ must have covered some previously unknown space that lies outside the frontier, $F_{t-1}$. At the same time, some part of $S\left(O_{t}\right)$ must lie inside known space, since the robot sensor must always reside in known space. $ℰ\left(O_{t}\right)$ must therefore intersect at least once with $F_{t-1}$ as shown in Figure 2C.

A set of cells can therefore be defined by $S\left(O_{t}\right)\setminus P_{t-1}^{\text{free}}$. This set will be surrounded by the subset of $F_{t-1}$ that lies inside $S\left(O_{t}\right)$, and the subset of $ℰ\left(O_{t}\right)$ that lies outside $P_{t-1}^{\text{free}}$.

FTFD involves a BFS along frontier cells and obstacle cells, starting with the cells in $F_{t-1}\cap^{\text{​}}S\left(O_{t}\right)$, and with a subset of cells in $ℰ\left(O_{t}\right)$. Since the cells in $F_{t-1}\cap^{\text{​}}S\left(O_{t}\right)$ will be connected to the set, $ℰ\left(O_{t}\right)\setminus \left(P_{t-1}^{\text{free}}\cup^{\text{​ }}F_{t-1}\right)$, then all freespace cells in $ℰ\left(O_{t}\right)$ will be visited by the BFS. Since $ℰ\left(O_{t}\right)$ contains all possible members of $F_{\text{new}}$, all cells $f\in F_{\text{new}}$ will be evaluated and determined to be frontiers.

Lemma 5.2. Suppose that c is a freespace cell that is not on the boundary between known and unknown space at t, then c will not be labeled as a frontier cell at t.

PROOF. This proof follows from Lemma 5.1. Let $O_{t}$ represent the observation taken at *t*, and let the cells covered by $O_{t}$ be denoted $S\left(O_{t}\right)$. Let $ℰ\left(O_{t}\right)$ be the cells that are in $S\left(O_{t}\right)$, and are adjacent to cells in the robot map that are not in $S\left(O_{t}\right)$. Let $F_{t-1}$ be the set of frontier cells in the robot map at t−1 which represent the boundary between known space, $P_{t-1}^{\text{free}}$, “inside” the boundary, and unknown space “outside” the boundary.

If *c* is not a frontier cell, there are two possible cases:

**Case 1.** *c* **is an obstacle cell.** Obstacle cells encountered by the BFS in FTFD are not labeled as frontiers, so *c* will not be labeled as a frontier cell.

**Case 2.** *c* **is a freespace cell.** Then *c* is either in $P_{t-1}^{\text{free}}$ or in $S\left(O_{t}\right)$, but it cannot be in $ℰ\left(O_{t}\right)\setminus \left(P_{t-1}^{\text{free}}\cup^{\text{​ }}F_{t-1}\right)$. This means *c* will not be evaluated by FTFD, and cannot be labeled as a frontier at *t*. If *c* was labeled a frontier at a previous timestep, then since it is in the active area, it is evaluated at *t* and removed from the set of frontiers.

### 5.2 FTFD Theoretical Analysis

At each iteration of FTFD, a constant-sized portion of the map is evaluated. The upper bound is set by the maximum area that can be covered by the sensor, $A_{\text{max}}$. The maximum difference in size between $F_{t-1}$ and $F_{t}$ is thus $A_{\text{max}}$, such that $F_{t-1}$ and $F_{t}$ can be considered interchangeable in terms of big-O notation.

#### 5.2.1 Finding Relevant Frontiers in $F_{t-1}$.

A key difference between previous frontier detection approaches and both EWFD and FTFD, is that EWFD and FTFD make use of the latest scan’s (or sequence of scans’) active area. This allows them both to find the relevant frontiers from the previous time step, and thus to detect new frontiers and remove old frontiers as needed at the current time step.

The active area of a scan is the set of cells that contains every cell that might have been affected by the scan. This set therefore also includes cells adjacent to those modified by the scan, since these might no longer have unknown neighbors, and will have lost their frontier status. For ease of computation, the active area can be estimated using a bounding box. The active area is used to bound all operations for detecting frontiers to the region that has changed as a result of the latest scan.

If the set of frontiers at timestep, t−1 is $F_{t-1}$, and the set of cells in the active area at time, *t* is $A_{t}$, then finding all the frontiers from the previous time step in the current scan’s (or sequence of scans’) active area can be efficiently performed using a range query on a balanced k-d tree (Kanth and Singh. (1997); Procopiuc et al., (2003)) in:

$$O({\left|F_{t-1}\right|}^{\left(d-1\right)/d}+\left|F_{t-1}\cap^{\text{​ }}A_{t}\right|),$$ (1)

where *d* is the dimensionality of the tuples. This is only a time-saving operation compared to evaluating all cells in $A_{t}$ if ${\left|F_{t-1}\right|}^{\left(d-1\right)/d}+\left|F_{t-1}\cap^{\text{​ }}A_{t}\left|\leq \right|A_{t}\right|$.

An understanding of what this means in practice can be best acquired through specific examples, shown in Table 2. In sum, it is unlikely that searching for the frontiers in the active area will take longer than searching through all the cells in the active area.

#### 5.2.2 Evaluating Cells for Frontier-Status

The BFS used by FTFD starts with the set $F_{t-1}\cap^{\text{​ }}A_{t}$ in its queue, plus the cells intersected by the endpoints of the rays used to integrate the latest scan into the map (at most $ℰ\left(O_{t}\right)$). It will evaluate all cells that are new frontiers or are obstacles. Since cells have a constant number of adjacent cells, the BFS runs in order:

$$O\left(\left|F_{t-1}\cap^{\text{​ }}A_{t}\left|+\right|\left(P_{t}^{\text{obs}}\cap^{\text{​ }}A_{t}\right)\cup^{\text{​ }}ℰ\left(O_{t}\right)\right|\right).$$ (2)

Since these are both smaller than the constant $A_{\text{max}}$, this can be simplified to O(1).

#### 5.2.3 Deleting Frontiers

At any iteration, the most frontier cells that need to be deleted is the set $F_{t-1}\cap^{\text{​ }}A_{t}$. These cells will need to have their status in the occupancy grid changed, be removed from the ordered list of tuples, and then be removed from the binary tree of frontier groups. This will run in order:

$$O\left(\overset{\text{delete from grid}}{\overset{︷}{\left|F_{t-1}\cap^{\text{​ }}A_{t}\right|}}+\overset{\text{delete from grid}}{\overset{︷}{\left|F_{t-1}\cap^{\text{​ }}A_{t}\left|\times \log\right|F_{t-1}\right|}}\right)=O\left(\left|F_{t-1}\cap^{\text{​ }}A_{t}\left|\times \log\right|F_{t-1}\right|\right)=O\left(\log\left|F_{t-1}\right|\right)$$ (3)

#### 5.2.4 Inserting Frontiers

The most cells needing to be inserted at timestep, *t* is $\left|ℰ\left(O_{t}\right)\right|$, so that the complexity of inserting is:

$$O\left(\left|Ε\left(O_{t}\right)\cap^{\text{​ }}\left(P_{t}^{free}\setminus P_{t-1}^{free}\right)\left|\times \log\right|F_{t-1}\right|\right)=O\left(\log\left|F_{t-1}\right|\right).$$ (4)

#### 5.2.5 Labeling Frontiers

Using the occupancy grid to represent edges of a graph, and using the tree of frontier cells as the list of vertices, a series of DFSs can be performed to determine the connected sets of frontier cells. This runs in order: $O\left(\left|F_{t}\right|\right)$.

The total complexity per iteration of FTFD is therefore:

$$O\left({\left|F_{t-1}\right|}^{\left(d-1\right)/d}+1+\log\left|F_{t-1}\left|+\log\right|F_{t-1}\left|+\right|F_{t}\right|\right)=O\left({\left|F_{t-1}\right|}^{\left(d-1\right)/d}+\log\left|F_{t-1}\left|+\right|F_{t}\right|\right)=O\left(\left|F_{t}\right|\right).$$ (5)

Over all iterations, in the worst case, $\left|F_{t}\right|$ increases by a maximum constant, $A_{\text{max}}$ at each timestep and in the best case, $\left|F_{t}\right|$ remains constant over time. Therefore, the FTFD upper and lower bounds are $O\left(t^{2}\right)$ and Ω(t), respectively.

## 6 Experimental Results

Three sets of experiments have been conducted using the same algorithmic implementation code, developed in MATLAB. Experiment one uses simulated data to test the relative efficiency of the algorithms when detecting exploration frontiers in a controlled, known, simulated environment. An image of a known map was used to emulate the Simultaneous Localization and Mapping (SLAM) process as a map is gradually constructed from a sequence of sensor observations. Experiment two uses Gazebo as the simulation engine environment and a ROS node in MATLAB to handle the calculation of the algorithms. In this experiment, the aim is to investigate the possible overhead in this system setup, and to test the stability and repeatability of the algorithms when they are fed simulated sensor data from Gazebo that mimics real-world scenarios. Experiment three was conducted in a 60 × 60 m real-world office environment at the University of Technology Sydney (UTS), where a MP-700 mobile robot equipped with a SICK-S300 laser scanner was manually controlled to construct a map. The software and system setup was similar to Experiment two, with most processes handled in ROS, and the algorithms implemented in a MATLAB ROS node. The MATLAB node handles the frontier detection calculations for each algorithm. The main difference is that in Experiment three, a real robot took the place of the Gazebo simulator used in Experiment two. For all scenarios, the calculation times per scan have been recorded alongside the total number of cells evaluated per scan. The algorithms compared are the Naïve approach, NaïveAA, WFD, WFD-INC*, EWFD, and the newly proposed FTFD.

The experiments have been conducted to investigate and validate the following ideas:

- Given a set of different frontier detection algorithms, investigate which algorithms perform more efficiently and effectively in several typical mobile robot scenarios, then highlight the differences, strengths, and weaknesses of all algorithms;
- As a robot explores an environment, the number of frontier cells tends to increase. Therefore, it is necessary to investigate what is the relationship between each algorithm’s calculation time and the number of frontier cells;
- Given that the experiments have been conducted both in simulation and on a real robot, investigate if there are relationships or contradictions between the results obtained from the simulated experiments and the real-world cases;
- Determine whether the MATLAB ROS node implementation is fit for purpose and can handle the task of frontier detection in real-time, (i.e. faster than sensor data can arrive) for all setups and with all algorithms in both real-world and simulation.

In all scenarios, the experiments were conducted 10 times for each algorithm, and the median time taken is shown. Since the algorithms are not running on a real-time OS, occasionally (but very infrequently) Linux system processes will cause one-off non-repeatable spikes in processing time, in the order of tens of milliseconds. Using the median values of 10 runs, this noise is filtered out. Finally, to display the graphs clearly without unnecessary empty spaces, the processing time for the first iteration, in which the system is being set up, was excluded for all algorithms. The details will be discussed further in the result section.

### 6.1 Experiment 1

**FIGURE 4:**
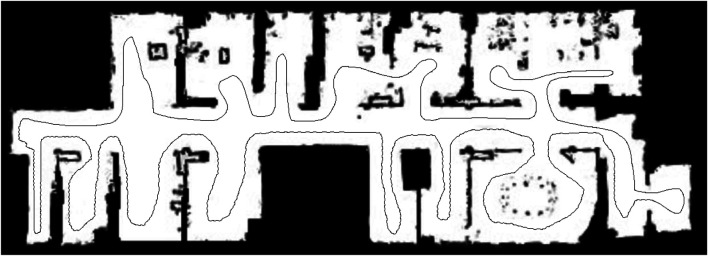
Freiburg lab environment with a prespecified trajectory for Experiment one.

The first experiment was conducted purely in MATLAB using a ground truth map image of a Freiburg lab environment of size 1,242 × 447 and shown in Figure 4 with a prespecified trajectory for a simulated robot to move through. At each point of the trajectory, a simulated laser scan was generated and ray-traced into the ground truth image. From that sensor observation, a local map was constructed to represent the current view of the simulated robot in the environment. The newly constructed map, and information about the active area of the latest sensor observation, (i.e. the bounding box of the latest scan) if relevant, was then put through each frontier detection algorithm to calculate and determine the frontier cells grouping.

The main purpose of this experiment is to verify and analyze the performance of each algorithm in perfect conditions with no delay due to information transmission between multiple computers, and no sensor noise.

The metrics measured for each algorithm were the average calculation time of each iteration, the total number of cells processed, and the total number of cells evaluated. The number of cells processed is defined as the number of cells being queried in some way, while evaluated means each time the algorithm checks whether a cell is a frontier or not.

Additionally, the NaïveAA, WFD-INC*, EWFD, FTFD algorithms all use the active area as part of the frontier detection. Therefore, the relationship between the size of the active area and the frontier calculation time is also evaluated by changing the maximum measuring range of the simulated laser scan. This value was increased gradually from 100 pixels to 500 pixels, with other parameters set to be the same for the 5 cases. The computational time for each case was then recorded.

### 6.2 Experiment 2

**FIGURE 5:**
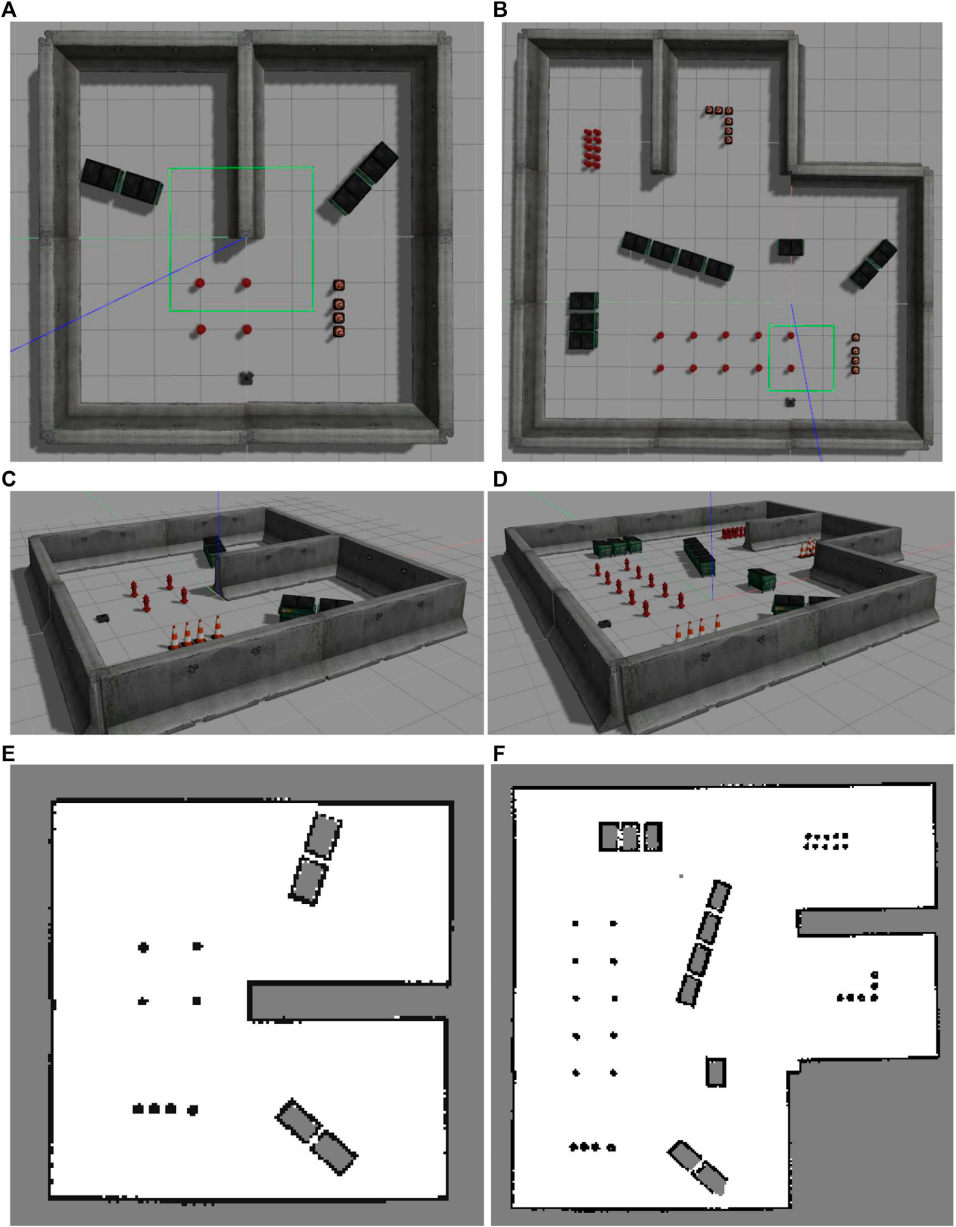
Two different map setups used in Experiment two. **(A)** Small map top view. **(B)** Large map top view. **(C)** Small map perspective view. **(D)** Large map perspective view. **(E)** Scanned small map. **(F)** Scanned large map.

**FIGURE 6:**
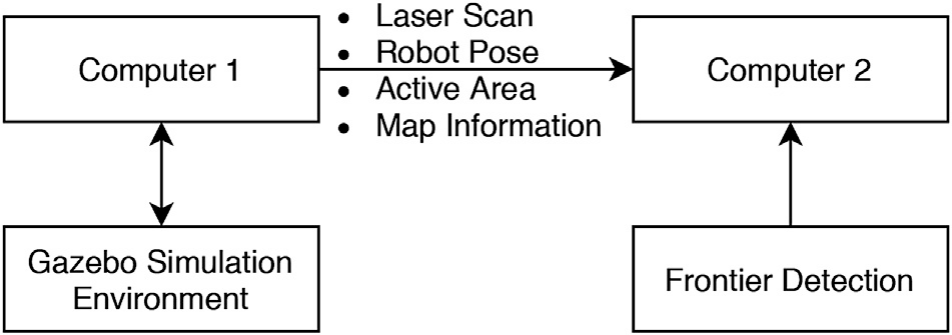
Experiment two setup.

The second experiment was conducted in two different environments as shown in Figure 5. Two different computers were used in the experimental system (Figure 6). The Gazebo simulator with the Turtlebot3 and the SLAM algorithm operated on the first computer. Meanwhile, the other computer subscribed to the data on the first one via a ROS network and performed frontier detection.

The Turtlebot3 was manually controlled to move around the simulated environment while the SLAM algorithm is also active. The map constructed by the SLAM algorithm for this experiment has a size of 20 by 20 m with a resolution of 0.05 m. The first computer synchronized the generated map and the necessary parameters such as the current robot pose, active area, and laser scan data and then published them through the ROS network. The second computer then collected the data and implemented them for each of the frontier detection algorithms in turn. To ensure a repeatable data set over multiple attempts in this experiment, the data from the first computer was recorded in a rosbag and then played back to all the frontier detection algorithms.

The main purpose of this simulated experiment is to validate the efficiency of the algorithms in a more realistic scenario where there exist delays caused by the SLAM algorithms and information transmission between different machines, and the sensor data and robot movement is similar to reality. This scenario also provides a basis for comparison with Experiment three, which will be mentioned in the following section, to validate the relationship between the simulation and real-world results.

### 6.3 Experiment 3

**FIGURE 7:**
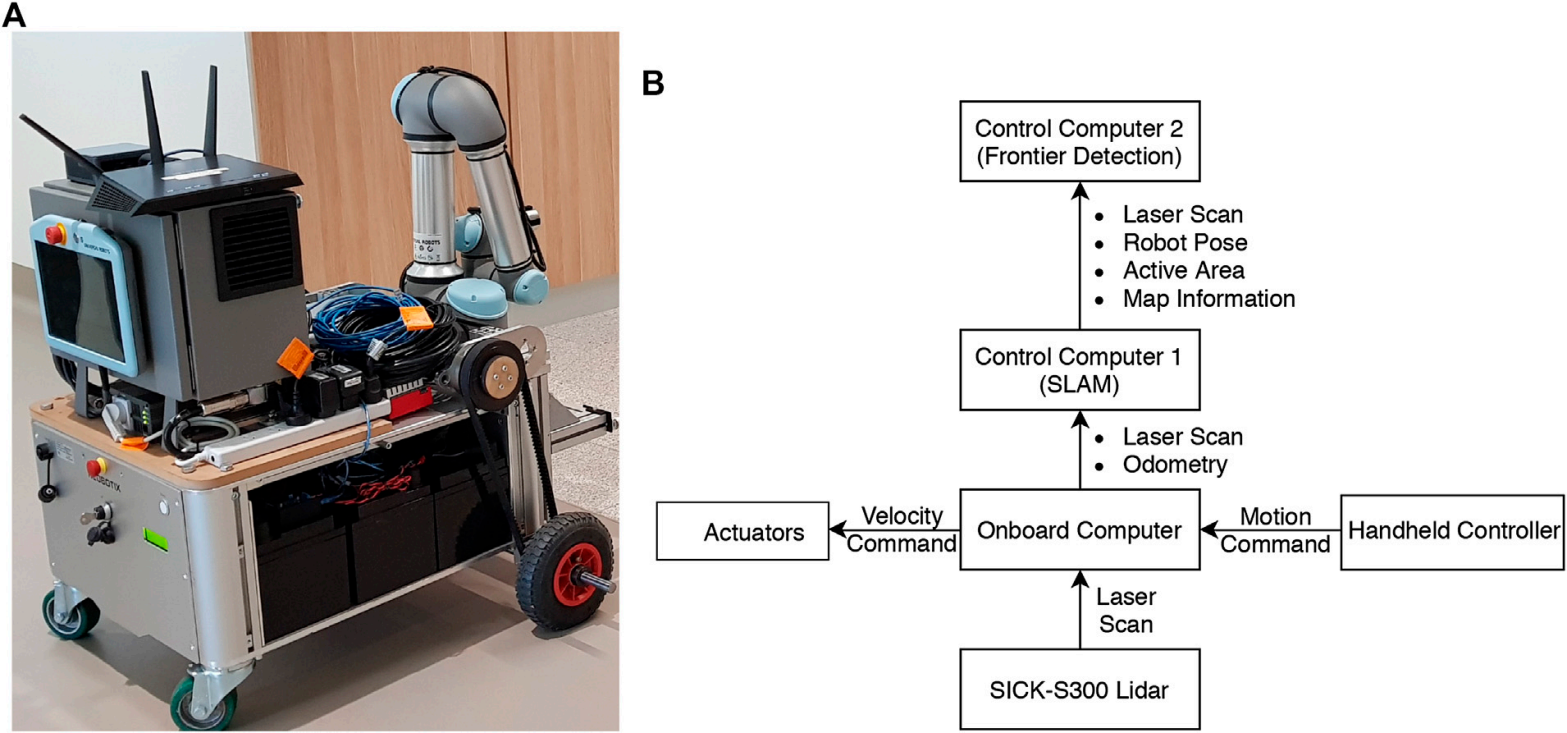
Experiment three real-world platform: **(A)** The Neobotix MP-700 mobile robot; **(B)** Robot and information diagram.

**FIGURE 8:**
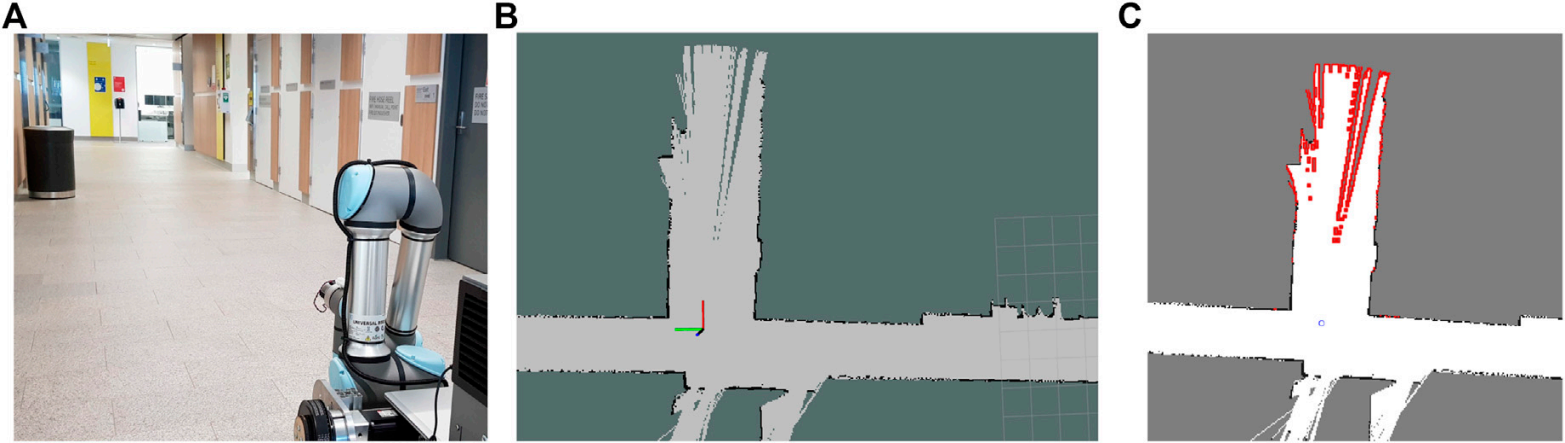
Experiment three setup. **(A)** Current real-world view of the robot. **(B)** Updating the map with the latest sensor observation (robot position indicated by frame annotation). **(C)** Newly detected frontiers (in red) in the constructed map.

The third experiment was conducted in the real-world using a Neobotix MP-700 mobile robot shown in Figure 7. The robot was driven manually controlled by a human operator around a large office environment inside UTS as shown in Figure 8. Laser scan data from a SICK LRF was collected and fused with odometry data from wheel encoders to incrementally build a map of the environment using ROS SLAM packages. Each scan was sent to the MATLAB ROS node to calculate the exploration frontiers by sequentially using the suite of detection algorithms. In order to have a consistent movement trajectory and a repeatable data set, the exploration frontiers calculation of each algorithm was performed on a previously recorded rosbag containing all the required parameters. The sensor mounted on the MP-700 robot has a field of view of 180° and a maximum range of 30 m. The map constructed by the SLAM algorithm for this scenario was limited to 200 by 200 m with a resolution of 0.05 m.

## 7 Results

**FIGURE 9:**
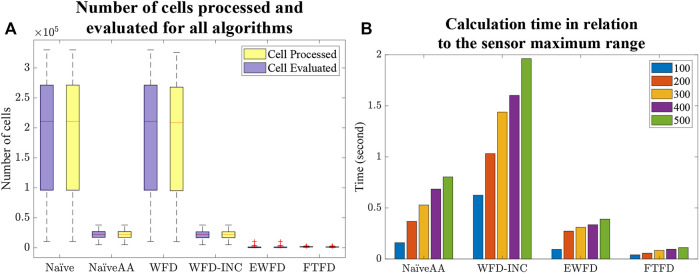
Experiment one results. **(A)** Number of cells processed and evaluated by all algorithms. **(B)** Calculation time of the algorithms that consider the active area in relation to the maximum range of the simulated sensor (measured in number of map cells).

**FIGURE 10:**
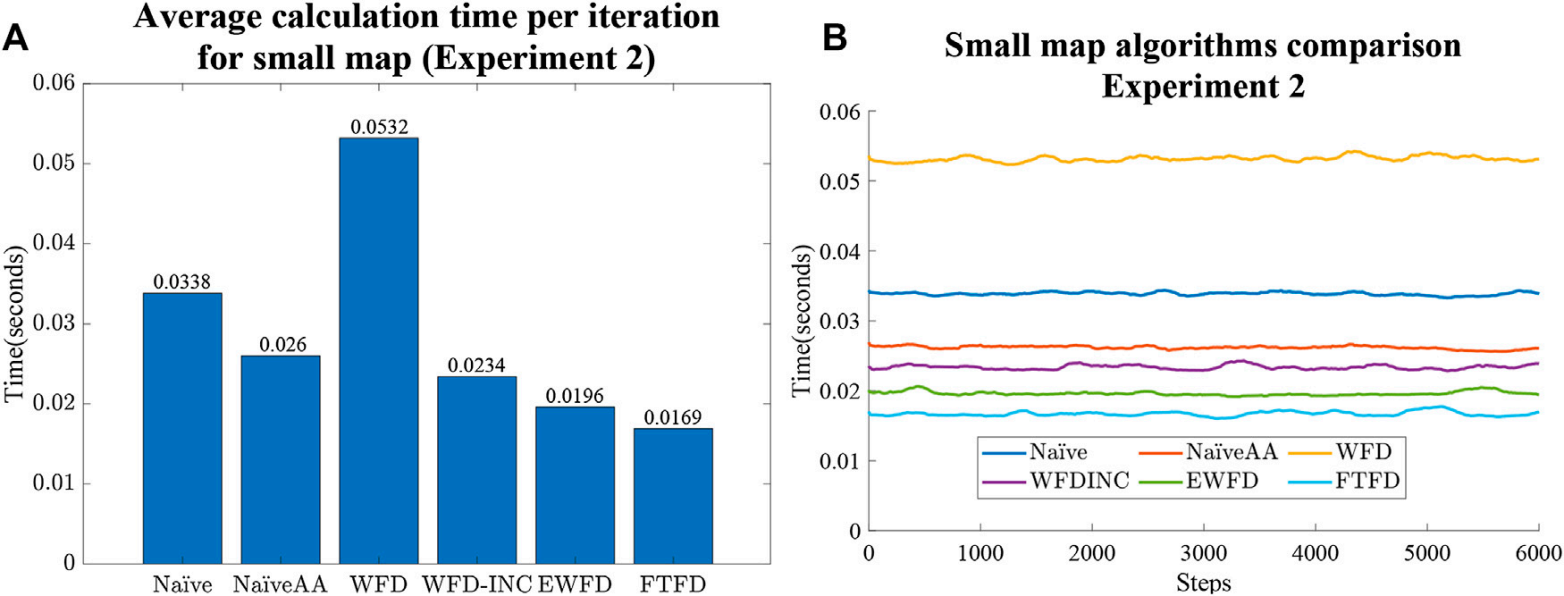
Experiment two results for the small map case. **(A)** Average calculation time per iteration. **(B)** Calculation time as the small map is gradually explored for all algorithms.

**FIGURE 11:**
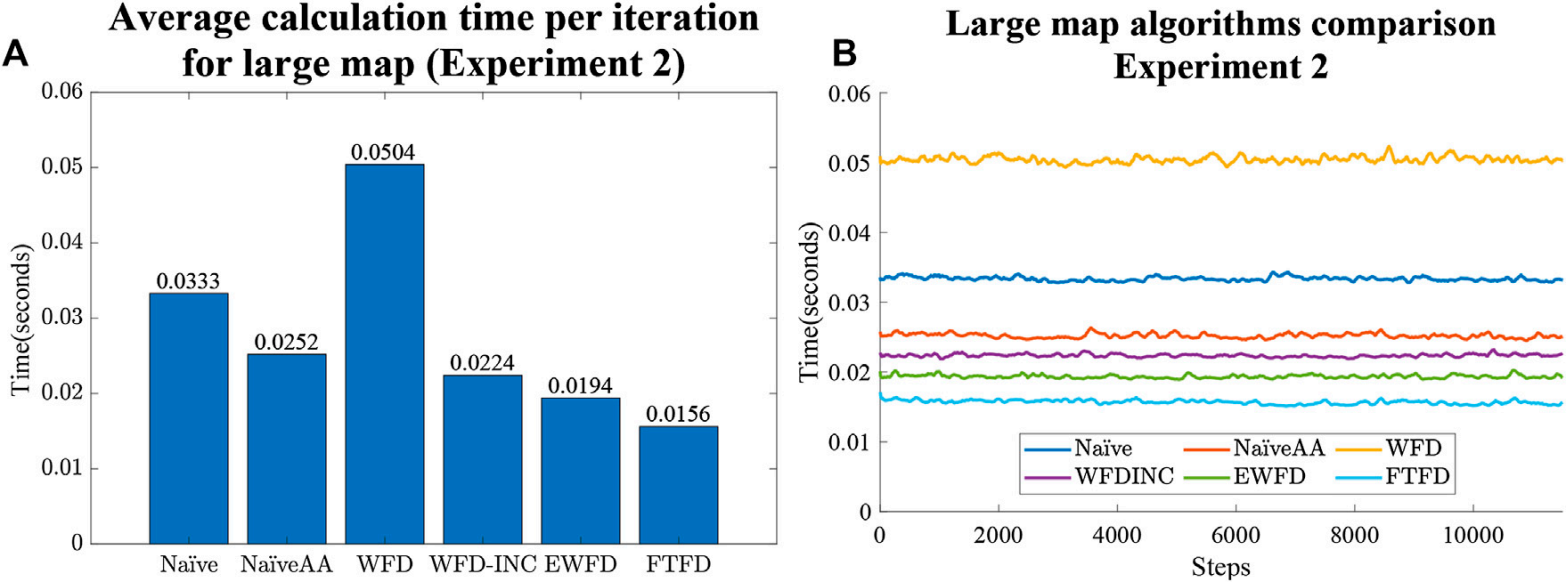
Experiment two results for large map case. **(A)** Average calculation time per iteration. **(B)** Calculation time as the large map is gradually explored for all algorithms.

First, from the Experiment one average calculation time shown in Figure 9A, it is clear that the number of cells processed and evaluated by both the Naïve and WFD algorithms are significantly higher than for the other algorithms. On the other hand, EWFD and FTFD are both drastically faster as they evaluate fewer cells than the other algorithms. This performance advantage will eventually result in the considerably lower overall processing time as shown Experiment two results for the small map (Figure 10) and the large map (Figure 11).

The Experiment one comparative plot of the sum of frontier detection time over all steps in the exploration (Figure 9B) demonstrates a strong relationship between the size of the active area and the total processing time for each of the algorithms in which the active area is considered. However, this relationship effect is smaller for EWFD and FTFD compared to the NaïveAA and WFD-INC*.

Both Figures 10A and 11B indicate that the average calculation time of each iteration of the NaïveAA, WFD-INC*, EWFD, and FTFD algorithms is lower than the update rate of 5 Hz used by the simulated SLAM algorithm. It is noted that 5 Hz is not the maximum rate possible, but is adequate for the robot speed in the experiments and can be guaranteed in all cases for all experiments. Since this research focuses on the frontier detection calculation time, the SLAM update and publish rate does not affect the results.

The processing time of both the Naïve and WFD algorithms is noticeably longer than other algorithms. Moreover, the novel approach, FTFD, produces a better overall result compared to the other approaches in terms of time and processed cells vs evaluated cells. Results from both the small and large maps in Experiment two (Figures 10B,11B) suggest that there is no relationship between the number of explored cells and the calculation time of each iteration on all algorithms since the calculation time for both map cases remains relatively stable as more sections of the map are revealed.

**FIGURE 12:**
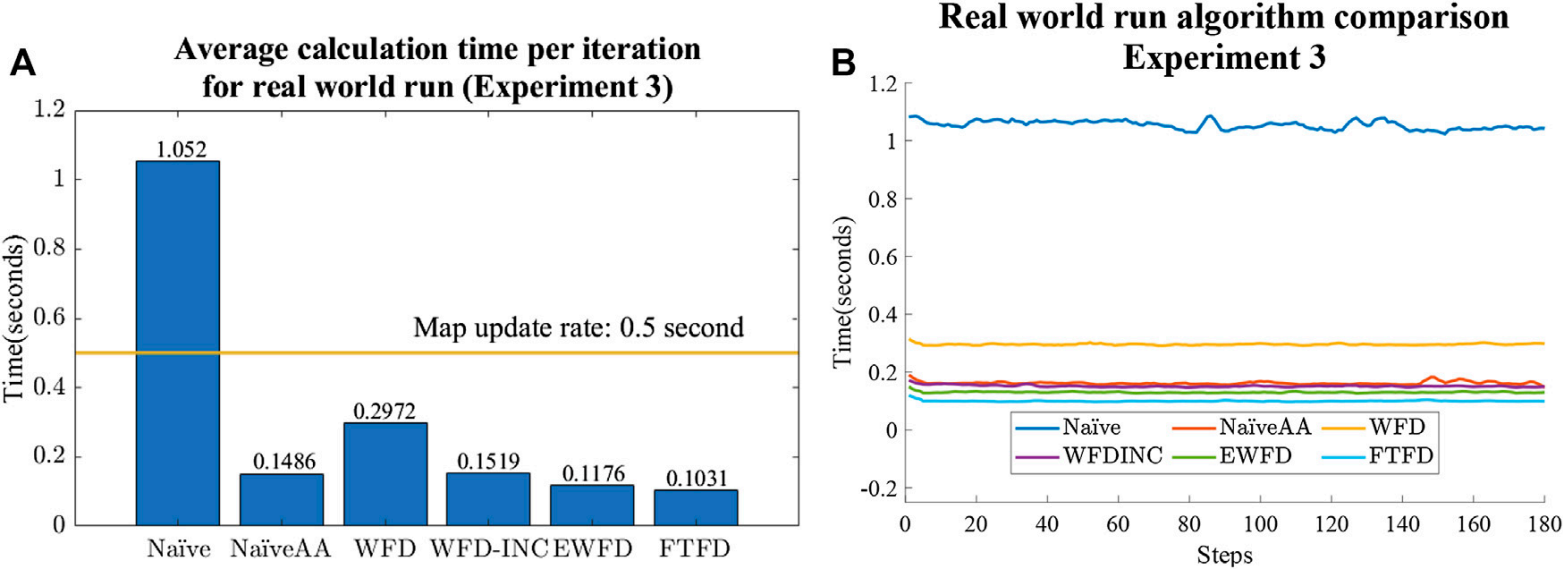
Experiment three results. **(A)** Average calculation time per iteration with a horizontal line representing the updating rate of the map constructed by the SLAM algorithm. **(B)** Calculation time changes as the map is explored for all algorithms.

Figures 12 and 10 illustrate the similarity between the results of the second and third experiments. In both experiments, EWFD and FTFD have the best performance, with the Naïve and WFD being the most time consuming of all of the algorithms. In the real-world Experiment three, as the map size is 200 by 200 m with a resolution of 0.05 m, the image of the map reaches 4,000 by 4,000 pixels, all algorithms exhibit a drop in performance compared to the Gazebo simulated Experiment two where the maps are smaller. However, most of the algorithms are significantly faster than the update rate of the SLAM algorithm, which is 2 Hz (0.5 s). The only exception is the Naïve algorithm, with an average calculation time of more than 1 s, which means it cannot keep up with the update rate.

**FIGURE 13:**
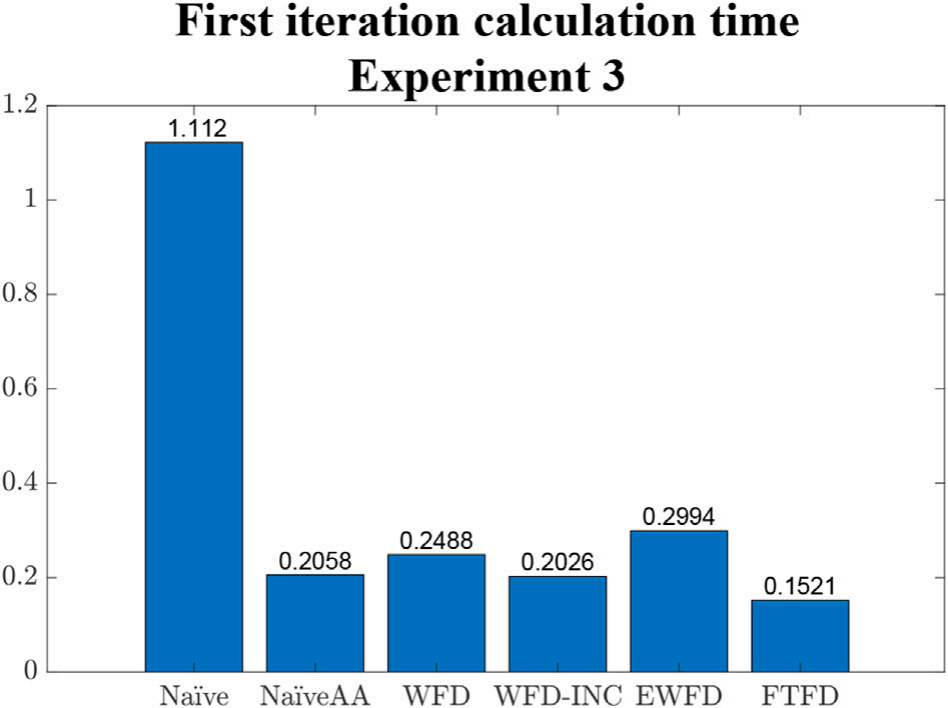
Average calculation time in only the **first** iteration of each algorithm.

Finally, the average calculation time for the first iteration of each algorithm is shown in Figure 13. Comparing with the results from Figure 12A, it is clear that most algorithms (except Naïve and WFD) required a relatively higher amount of time to set up the initial variables and, in the case of both EWFD and FTFD, to locate the first set of frontier cells.

## 8 Discussion

The results obtained from the different experiments provide insights into the overall performance of the different frontier detection algorithms. Importantly, it is clear that the results from both simulation Experiments (1 and 2) and the real-world Experiment three are in agreement. Therefore, it is appropriate to synthesize these results and comparatively discuss and highlight the algorithms’ strengths and weaknesses. A summary of properties and suggestions for each algorithm is shown in Table 3. It is noted that while all the algorithms can be run in 3D, their relative effectiveness has not been tested on 3D data within the scope of this paper.

**TABLE 2:** Minimum sizes of $F_{t}$ for $\left|A_{t}\right|<{\left|F_{t-1}\right|}^{\left(d-1\right)/d}$ to be true, based on two different sensors (Voxel resolution of 0.05m).

FOV	Max	≈Vol./	Voxels/Cells	$\left|F_{t}\right|$	$F_{t}$ Vol./
	Range	Area	In $A_{t})$		Area
$43^{\circ }$	2m	$1.05m^{3}$	$8.4\times 10^{3}$	$7.7\times 10^{5}$	$96m^{3}$
If bounding		$3.16m^{3}$	$2.5\times 10^{4}$	$4.0\times 10^{6}$	$502m^{3}$
Box used					
$180^{\circ }$	2m	$6.28m^{2}$	$2.5\times 10^{3}$	$6.3\times 10^{6}$	$15,775m^{2}$

**TABLE 3:** Properties and suggestions for using different frontier detection algorithms. Yes* is used to indicate where one algorithm performs markedly better than the others for a particular property.

Properties	Naïve	NaïveAA	WFD	WFD-INC	EWFD	FTFD
Performance stable as map size grows	No	No	Yes	Yes	Yes	Yes
Faster when run after no map change	No	No	No	No	Yes	Yes
Performance stable as active area increases	N/A	No	N/A	No	Yes	Yes*
Capable of detecting pockets	Yes	Yes	Yes	Yes	No	No
Ease of software implementation	Yes*	Yes	No	No	No	No
Must run after every scan	No	No	No	No	No	Yes
High update rate	No	No	No	No	Yes	Yes*
Suitable for open-spaced map	No	Not ideal	No	No	Not ideal	Yes
Suitable for maps with multiple narrow paths or corridors	No	Not ideal	No	No	Yes	Yes
Ideal for maps with low resolutions	No	Yes	No	Yes	Yes	Yes
Suitable for maps with high resolutions	No	No	No	No	Yes	Yes*

First of all, it is reasonable to conclude that there is a close relationship between the number of cells processed and evaluated and the computational time. Therefore, as the map either becomes more detailed, (i.e. high-resolution maps) or the sensor covers more area at the same time (i.e. large active area), algorithms that evaluate the smallest number of cells should be prioritized, such as the newly proposed Frontier-Tracing Frontier Detection (FTFD) or Expanding-Wavefront Frontier Detection (EWFD).

However, considering the complexity of implementing both EWFD and FTFD, in scenarios where the resolution of the constructed map is relatively low, or the sensor’s maximum range is short, Naïve Active Area should be considered, due to its simplicity in implementation while also being able to yield similar performance, comparing with both EWFD and FTFD.

**FIGURE 14:**
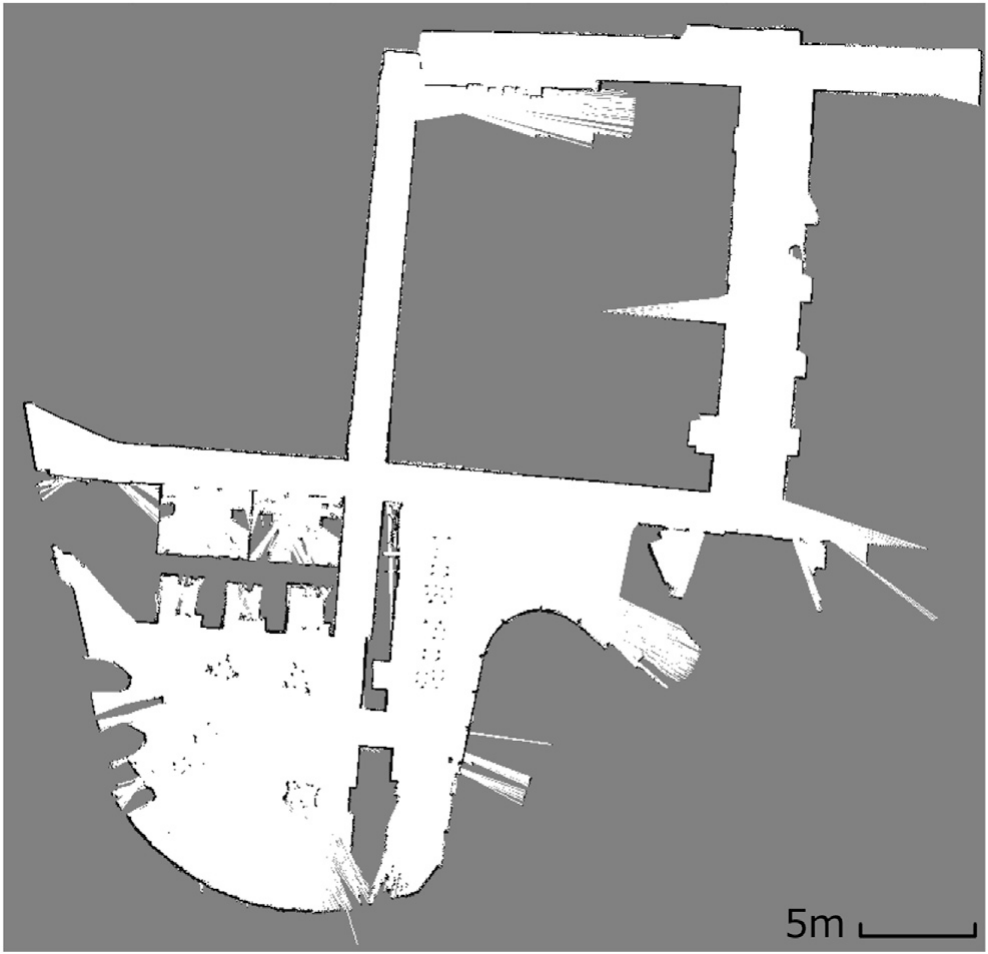
Scanned map of Experiment three.

Secondly, when the map contains large open areas, (e.g. Experiment two maps - Figure 5), FTFD outperforms EWFD clearly as FTFD only evaluates the perimeter of each laser scan while EWFD processes the area. However, if the map contains multiple corridors or narrow paths, (e.g. Experiment three map - Figure 14), then EWFD is comparatively similar to FTFD, as the number of cells covered by the scan area and perimeter are now more equal.

Thirdly, as shown in Figures 10B and 11B, there is no significant relationship between frontier detection calculation time and the expansion of the map, (i.e the discovery of more free space). However, in theory, more free cells should mean an increase in processing time as there are more cells to be evaluated.

Finally, the results confirm that Naïve Active Area should be used as a benchmark for all future frontier detection algorithm for its simplicity, robustness, and efficiency, compared to Naïve frontier detection, whose performance degrades with the size of the map. Furthermore, when it comes to performing frontier detection after each observation (which is common practice in exploration tasks), FTFD should be considered, as it functions most effectively after each laser scan and is also the algorithm with the highest efficiency among those evaluated. Additionally, as shown in Figure 12, it is reasonable to conclude that the MATLAB ROS Node is suitable for handling the task of frontier detection, since all algorithms in all real and simulated maps were able to calculate the frontier cells within the map update rate of the SLAM algorithm, other than Naïve in the largest real-world map.

## 9 Conclusion

This paper proposed a novel approach to the problem of frontier detection by introducing the Frontier-Tracing Frontier Detection (FTFD) algorithm, while also providing comparisons between this novel approach and a few notable frontier detection algorithms. Simulations, along with real-world experiments, were conducted to provide insightful conclusions to the performance of the proposed algorithm, in comparison with the other approaches. The FTFD approach utilizes the perimeter of the current sensor observation in combination with the laser scan endpoints and the previously known frontiers to perform frontier detection. To validate the efficiency of FTFD and other approaches, three different experiments were implemented with relevant data collection for the overall computational time, the number of cells processed and evaluated, etc. From the results obtained, the insights can be summarized as follows. First of all, it is recommended that the frontier detection algorithm should be chosen based on the properties of the map and whether the speed of frontier detection is a crucial factor for the current application. More suggestions on which algorithm should be used for different cases are summarized in Table 3, based on the behaviors and properties of each algorithm. Results did not show a clear relationship in computational time and the exploration of the map for any algorithm. In other words, more free space does not proportionally increase the overall calculation time for the Naïve, NaïveAA algorithms. Finally, NaïveAA should be considered as a new benchmark for evaluating future frontier detection algorithm due to its speed, robustness, and efficiency over Naïve frontier detection, while FTFD is best suited for applications that require frontier detection to be implemented after each observation.

Though the presented algorithms will work in 3D, it is not immediately obvious how much advantage EWFD and FTFD would provide compared to other algorithms when used in 3D environments. Further experiments using 3D data still need to be performed in future. The authors also intend to test each frontier detection algorithm when used as part of a holistic robot exploration framework. This will allow an evaluation of the effects, if any, that shorter computation times and higher rates of frontier detection may have on the behavior and efficiency of autonomous exploration. Also, it would be interesting to determine whether dynamically selecting between available frontier detection algorithms, depending on the situation, can further improve results.

## Data Availability

The raw data supporting the conclusion of this article will be made available by the authors, without undue reservation.

## References

[B1] Digor E. Birk A. Nüchter A. (2010). Exploration strategies for a robot with a continously rotating 3D scanner. Proceedings of the Second International Conference on Simulation, modeling, and programming for autonomous robots, SIMPAR’10, Darmstadt, Germany, 15–18 November, 2010. Berlin, Germany: Springer-Verlag, 374–386. 10.1007/978-3-642-17319-6_35

[B2] Dornhege C. Kleiner A. (2011). A frontier-void-based approach for autonomous exploration in 3D. In 2011 IEEE International Symposium on Safety, Security, and Rescue Robotics (SSRR), Kyoto, Japan, 1–5 November, 2011, 351–356. 10.1109/SSRR.2011.6106778

[B3] Faigl J. Kulich M. (2013). On determination of goal candidates in frontier-based multi-robot exploration. 2013 European Conference on Mobile Robots (ECMR), Barcelona, Spain, 25-27 Sept. 2013, 210–215. 10.1109/ECMR.2013.6698844

[B4] Hassan M. Liu D. Paul G. (2018). Collaboration of multiple autonomous industrial robots through optimal base placements. J. Intell. Rob. Syst. 90, 113–132. 10.1007/s10846-017-0647-x

[B5] Hornung A. Wurm K. M. Bennewitz M. Stachniss C. Burgard W. (2013). OctoMap: an efficient probabilistic 3D mapping framework based on octrees. Aut. Robots 34, 189. 10.1007/s10514-012-9321-0

[B6] Kanth K. V. R. Singh A. K. (1997). Optimal dynamic range searching in non-replicating index structures. In Proceedings of the International Conference on Database Theory, Delphi, Greece, 8–10 January, 1997. 257–276. 10.5555/645503.656269

[B7] Keidar M. Kaminka G. A. (2014). Efficient Frontier detection for robot exploration. Int. J. Robot Res. 33, 215–236. 10.1177/0278364913494911

[B8] Keidar M. Kaminka G. A. (2012). Robot exploration with fast Frontier detection: theory and experiments. Proceedings of the 11th International Conference on Autonomous Agents and Multiagent Systems—Volume, Valencia, Spain, June, 2012, 1, 113–120. 10.5555/2343576.2343592

[B9] Liu L. Fryc S. Wu L. Vu T. Paul G. Vidal-Calleja T. (2020). Active and interactive mapping with dynamic Gaussian process implicit surfaces for mobile manipulators https://arxiv.org/abs/2010.13108.

[B10] Orsulic J. Miklic D. Kovacic Z. (2019). Efficient dense Frontier detection for 2-d graph slam based on occupancy grid submaps. IEEE Robot. Autom. Lett. 4, 3569–3576. 10.1109/lra.2019.2928203

[B11] Paul G. Liu L. Liu D. (2016). A novel approach to steel rivet detection in poorly illuminated steel structural environments. 2016 14th International Conference on Control, Automation, Robotics and Vision (ICARCV), Phuket, Thailand, 13-15 November, 2016, 1–7. 10.1109/ICARCV.2016.7838630

[B12] Paul G. Quin P. To A. W. K. Liu D. (2015). A sliding window approach to exploration for 3d map building using a biologically inspired bridge inspection robot. 2015 IEEE International Conference on Cyber Technology in Automation, Control, and Intelligent Systems (CYBER), Shenyang, China, 8-12 June 2015. 1097–1102. 10.1109/CYBER.2015.7288098

[B13] Procopiuc O. Agarwal P. Arge L. Vitter J. (2003). Bkd-tree: a dynamic scalable kd-tree. Advances in spatial and temporal databases, Berlin Germany: Springer, 2750, 46–65. 10.1007/978-3-540-45072-6_4

[B14] Qiao W. Fang Z. Si B. (2018). Sample-based Frontier detection for autonomous robot exploration. 2018 IEEE International Conference on Robotics and Biomimetics (ROBIO), Kuala Lumpur, Malaysia, 12-15 Dec. 2018, 1165–1170. 10.1109/ROBIO.2018.8665066

[B15] Quin P. Alempijevic A. Paul G. Liu D. (2014). Expanding wavefront Frontier detection: an approach for efficiently detecting Frontier cells. Proceedings of Australasian Conference on Robotics and Automation, Melbourne, Australia, December 2014.

[B16] Quin P. Paul G. Alempijevic A. Liu D. (2016). Exploring in 3d with a climbing robot: selecting the next best base position on arbitrarily-oriented surfaces. 2016 IEEE/RSJ International Conference on Intelligent Robots and Systems (IROS), Daejeon, South Korea, 9-14 October 2016, 5770–5775. 10.1109/IROS.2016.7759849

[B17] Quin P. Paul G. Alempijevic A. Liu D. K. Dissanayake G. (2013). Efficient neighbourhood-based information gain approach for exploration of complex 3d environments. Proceedings of IEEE International Conference on Robotics and Automation (ICRA), Karlsruhe, Germany, 6-10 May 2013, 1335–1340. 10.1109/ICRA.2013.6630745

[B18] Quin P. Paul G. Liu D. (2017). Experimental evaluation of nearest neighbor exploration approach in field environments. IEEE Trans. Autom. Sci. Eng. 14, 869–880. 10.1109/tase.2016.2640228

[B19] Reid R. Cann A. Meiklejohn C. Poli L. Boeing A. Braunl T. (2013). Cooperative multi-robot navigation, exploration, mapping and object detection with ros. 2013 IEEE Intelligent Vehicles Symposium IV, Gold Coast, QLD, Australia, 23-26 June 2013, 1083–1088. 10.1109/IVS.2013.6629610

[B20] Senarathne P. G. C. N. Wang D. (2015). Incremental algorithms for safe and reachable Frontier detection for robot exploration. Robot. Autonom. Syst. 72, 189–206. 10.1016/j.robot.2015.05.009

[B21] Senarathne P. Wang D. Wang Z. Chen Q. (2013). Efficient Frontier detection and management for robot exploration. 2013 IEEE International Conference on Cyber Technology in Automation, Control and Intelligent Systems, Nanjing, China, 26-29 May 2013, 114–119. 10.1109/CYBER.2013.6705430

[B22] Shade R. Newman P. (2011). Choosing where to go: complete 3D exploration with stereo. Proceedings of the IEEE International Conference on Robotics and Automation (ICRA), Shanghai, China, 9–13 May 2011, 2806–2811. 10.1109/ICRA.2011.5980121

[B23] Twigg J. N. Fink J. R. Yu P. L. Sadler B. M. (2013). Efficient base station connectivity area discovery. Int. J. Robot Res. 32, 1398–1410. 10.1177/0278364913488634

[B24] Wettach J. Berns K. (2010). Dynamic Frontier based exploration with a mobile indoor robot. Robotics (ISR), 41st International Symposium on and 6th German Conference on Robotics (ROBOTIK), Munich, Germany, 7–9 June 2010, 1–8.

[B25] Wurm K. M. Hornung A. Bennewitz M. Stachniss C. Burgard W. (2010). OctoMap: a probabilistic, flexible, and compact 3D map representation for robotic systems. Proceedings of the ICRA 2010 workshop, Anchorage, AK, United States, January 2010.

[B26] Yamauchi B. (1997). A frontier-based approach for autonomous exploration. In. Proceedings 1997 IEEE International Symposium on Computational Intelligence in Robotics and Automation CIRA'97. “Towards New Computational Principles for Robotics and Automation”, Monterey, CA, United States, 10-11 July 1997, 146–151. 10.1109/CIRA.1997.613851

